# Breaking Barriers: The Design and Development of an Assistive Technology Web App for Older Latinos with Disabilities in Daily Activities

**DOI:** 10.3390/technologies12110232

**Published:** 2024-11-19

**Authors:** Elsa M. Orellano-Colón, Adriana I. Ramos-Marichal, Valeria R. González-Crespo, Bianca N. Zeballos-Hernández, Kenneth N. Ruiz-Márquez, Abiel Roche-Lima, Joan M. Adorno-Mercado, Norman A. Laborde-Torres, Joshua G. Berríos-Llopart, Angely M. Cruz-Ramos, Dana V. Montenegro, Carmen E. Lamoutte, Natasha D. Rosa-Casilla, David E. Meléndez-Berrios

**Affiliations:** 1 School of Health Professions, University of Puerto Rico Medical Sciences Campus, San Juan 365067, Puerto Rico; 2 Research Centers in Minority Institutions (RCMI) Center for Collaborative Research in Health Disparities, University of Puerto Rico Medical Sciences Campus, San Juan 365067, Puerto Rico; 3 Wovenware A Maxar Company, San Juan 00909, Puerto Rico

**Keywords:** activities of daily living, assistive technology, Latinos, mHealth, older adults, self-management, web application

## Abstract

Latinos are among the populations who are the least likely to use assistive technology (AT) despite being a population with a high prevalence of functional disabilities (FDs). We aimed to create and test the usability of an AT web app for independent-living older adults with FDs. In Phase I, we created the web app’s content guided by the Optimized Honeycomb Model and considered the AT needs and FDs of older Puerto Ricans found in our previous studies. In Phase II, we design the web application by adopting a Lean UX process and design heuristics for older adults. In Phase III, we conducted usability testing using focus groups and individual interviews with 14 older adults, interpreted through a directed content analysis. The Mi Guía de Asistencia Tecnológica (MGAT) was developed with ninety-four AT devices in eight areas of daily activities. The MGAT provides comprehensive information on AT, including photos and videos of older adults using AT. Participants reported that the MGAT was usable, accessible, credible, desirable, useful, and valuable in increasing their knowledge of AT. These findings are a foundation for developing efficient AT information strategies using such technology as a first step to improving AT adoption among older adults.

## 1. Introduction

Functional disabilities (FDs) are any difficulty in performing or participating in daily activities necessary for independent living, such as walking, bathing, or completing household tasks [1]. FDs can be a result of the normal aging process or chronic conditions related to age, such as arthritis, diabetes, hypertension, stroke, heart disease, emphysema/chronic obstructive pulmonary disease, cognitive impairments, or cancer [2]. FDs are a significant public health problem, disproportionately affecting older adults in the continental United States (U.S.) and Puerto Rico. National reports show that the percentage of older adults (65 years and older) with disabilities in daily activities is considerably higher among older Latinos living in Puerto Rico (27.6%) compared to older adults living in the US (13.8%) [3]. The higher disability rates among older adults in Puerto Rico increase their vulnerability to experiencing health problems at individual and societal levels. At the individual level, FDs decrease the ability to perform and participate in activities of daily living (ADL), encompassing fundamental self-care tasks such as bathing, dressing, and eating [4]. Additionally, they impede instrumental activities of daily living (IADL), which involve the more complex tasks necessary for independent living, including managing finances, cooking, and transportation [4]. These limitations among older adults result in increased dependence, decreased quality of life, poor self-efficacy, fear of falling, social isolation, depression, and poor levels of health [5,6]. At the societal level, FDs increase the demand for healthcare services, resulting in higher medical spending [7].

Since function in activities of daily life is an essential prerequisite for healthy aging [8], addressing the functional needs of older adults is a significant public health priority. Assistive technology (AT) devices, which encompass a wide range of items, equipment, or product systems designed to enhance the functional abilities of people with disabilities [9], are crucial in promoting functional independence among older adults. These devices include low-tech options like jar openers, shower benches, and mobility aids. By directly addressing physical limitations, AT devices empower older people to engage in meaningful activities and occupations [10–13]. Utilizing assistive technology (AT) devices can decrease the likelihood of institutionalization, lower healthcare service costs, and enhance the safety, well-being, and overall quality of life for this population. [12]. On the contrary, the lack of use of AT devices among older adults can result in poor health and limitations in community participation [14].

Despite the benefits of using AT devices, older Latinos are the least likely to use and access AT devices [15]. Barriers to AT use among older Latinos with FDs living in Puerto Rico include the lack of information and knowledge regarding the existence of AT devices and services, the complexity or difficulty of understanding them, concerns about their safety, poor attractive appearance, bureaucracy on the acquisition process, social and cultural stigma, and a lack of prescription by healthcare professionals [16]. In a study with a similar sample, additional barriers related to equipment availability, discomfort with the use of technology, lack of functional needs, personal preferences (use of other strategies or help from others), and limited access to AT services were reported [17]. Among these, the lack of AT knowledge and information on AT devices and services was the most frequently reported barrier to accessing and using AT. Addressing these barriers is vital, as research has documented that information on the purchase of AT devices is the most essential prerequisite to using AT [18].

Typically, older adults with physical FDs living in Puerto Rico who gain access to low-tech AT devices and services receive institutional rehabilitation services for health conditions such as stroke, knee or hip replacements, traumatic brain injury, or cardiopulmonary diseases. Currently, interventions that address the AT needs of older adults living in the community with physical function disabilities due to chronic diseases but without acute episodes of health conditions are rare. Two main contributors to this service gap are the shortage of community occupational therapists in Puerto Rico and the lack of attention provided by primary healthcare providers to older adults with FDs. If FDs in this population are left unattended, the functional deterioration of this vulnerable group will continue to escalate, increasing the risk of early institutionalization and death.

Mobile health technologies (mHealth) refer to the use of mobile devices (such as smartphones, tablets, and wearable devices) and associated applications (apps) to support and deliver health-related services, information, and resources [19]. mHealth includes mobile apps as well as web apps. Mobile applications must be downloaded and installed from app stores, are platform-specific, run directly on a user’s device, and offer offline functionality. On the other hand, web applications are accessed through web browsers and do not require installation. Some of the advantages of web apps over mobile apps are the following: (1) they work on various devices and platforms without separate versions; (2) they are accessible across various devices and platforms; and (3) they are cost-effective, they are easier to maintain, and updates are immediately available to all users [20]. In addition, web applications can be designed to meet accessibility standards, are easily shareable via URLs, have the potential for a wider market reach, and can be indexed by search engines. However, they need an Internet connection to function and do not have access to device features.

In Puerto Rico, the urban and suburban areas where most older adults live generally enjoy good internet access. Additionally, the federal and local governments have launched initiatives to improve internet access on the island of Puerto Rico. However, the internet connection could be a specific challenge faced by older people living in rural areas of the island because infrastructure development in these regions is slower and high-speed options such as fiber optics are rarely available. In addition, the limited digital literacy of older people with low educational levels makes it difficult for them to use the Internet even when it is available effectively. Despite these challenges, mHealth mobile and web apps of mHealth have great potential to provide more efficient healthcare to people with limited resources, especially older adults [21]. Therefore, mHealth apps have been developed for various medical conditions, such as diabetes [22], severe mental illness [23], cancer [24], and stroke [25]. However, very few of these applications have been empirically developed [23–25], and there is a paucity of applications that target low-tech AT devices for older adults with physical FDs. In this study, we aimed to develop an AT guide web app tailored for older Latinos living in the community facing physical FDs in ADLs and IADLs. Our specific objectives were to (1) determine the evidence-based content of the AT guide web app; (2) design the AT guide web app; (3) describe how older Latinos use, think, and feel about the AT guide web app; and (4) identify older Latino recommendations for improving the design of the AT guide web app.

The primary contribution and originality of the AT guide web app prototype lie in its targeted focus on providing comprehensive information on assistive technology (AT) devices and resources specifically designed for Latino older adults with physical functional disabilities (FDs). This tailored content is vital, as it encompasses a wide array of AT devices and resources, detailing their features, benefits, availability, and instructional videos which showcase older adults using these devices. This represents a significant advancement in the field, especially considering that existing resources, such as Atvisor.ai, [26], primarily offer general descriptions of various AT devices, their costs, and acquisition options. This resource is limited because it does not specifically target the older adult population and lacks video support to help them to learn to use assistive technology devices (AT).

In addition, the findings of this prototype can act as a catalyst for future initiatives aimed at promoting the adoption of AT devices through accessible platforms, specifically targeting the vulnerable population of older Hispanics. This strategic approach is a crucial step toward bridging the disparities in the prevalence of functional disabilities, ultimately improving the quality of life for this underserved community.

## 2. Materials and Methods

This study was approved and conducted according to the ethical guidelines established by the Institutional Review Board of the University of Puerto Rico Medical Sciences Campus (Protocol A4120121). We used a qualitative research design by incorporating older Latinos’ input through focus groups and individual interviews [27]. Karagianni’s Optimized Honeycomb Model [28], initially developed by Morville et al. (UX Honeycomb) [29], was used to guide the design of the AT guide web app, obtain and analyze the user experience perspective, and evaluate the effectiveness of the application to accommodate the needs of older adults. This model includes seven facets grouped into three dimensions to explore how the users ‘Use’ (Findable; Usable; Accessible), ‘Think’ (Desirable; Credible), and ‘Feel’ (Useful; Valuable) about an application [28]. This study was conducted in three phases: (1) Phase I consisted of determining the content of the AT guide web app; (2) Phase II consisted of the development of the design features of the AT guide web app; and (3) Phase III consisted of the usability testing of the AT guide web app using focus groups and individual interviews.

### 2.1. Phase I: Determining the Content of the AT Guide App

The content of the web app for the AT guide was determined based on the evidence of the need for AT devices in older Latinos living in Puerto Rico and the barriers to the use of AT devices in this population found in our previous studies [16,17,30]. The first descriptive study was conducted with a purposive sample of 60 independent living Hispanics 70 years or older from urban and rural communities on the island of Puerto Rico, with a physical function disability and no cognitive impairment. These participants reported among 50 AT devices for ADLs and IADLs and categorized them into devices they used and devices they did not use but were willing to use [30]. The second cross-sectional study was conducted with 211 older Latinos 65 years or older who lived independently and did not have cognitive impairments or functional disabilities and who were randomly recruited from poor communities in San Juan, Puerto Rico. This study identified the most prevalent physical disabilities using the PROMIS Physical Function Short Form [31]. Two additional qualitative studies conducted with the purposive sample [16] and the randomly selected sample [17] described above identified the barriers to using AT devices among eighty-three older Latinos. Modifiable barriers to the use of AT were considered in the selection of AT devices and the selection of the content associated with each AT device.

#### 2.1.1. Data Collection Methods

Three tables were created to determine the content of the AT guide web app. Table 1 classifies the 41 AT devices that compensate for physical FDs reported in the Orellano-Colón et al. 2018 study [30]. AT devices are organized in rows and classified as ‘I already have this’ and ‘I would use this but don’t have it’. The final column reports our decisions on including the AT device in the AT guide web app. Table 2 pairs the frequency of some level (little, some, or much) of physical FDs in IADLs, functional mobility, and self-care activities (organized in rows), as reported by the PROMIS v1.2 Physical Function 20a Spanish, with AT devices that could compensate for these disabilities (organized in columns) [31]. The final column reports the researchers’ decisions regarding including the AT device in the AT guide web app. Table 3 reviews the alignment of the modifiable barriers to the use of AT devices among older Latinos [16,17] with the strategy used in the web app of the AT guide to address each barrier.

#### 2.1.2. Procedures

Our research team met twice to assess and discuss the AT devices that emerged from the AT needs of 60 older Latinos and the physical FDs reported by 211 older Latinos in our previous studies, which are included in Tables 1 and 2. These discussions led to a consensus on the final decision on whether or not to include AT devices in the AT guide web app content. Subsequently, the researchers met again to discuss and reach a consensus on what barriers to using AT devices would be addressed by the AT guide web app and how this application would address the selected barriers.

#### 2.1.3. Data Analysis

The identified assistive devices were analyzed based on the frequency at which an AT device was reported for ADLs or IADLs as needed and the frequency of the functional needs reported in our previous studies. We determined whether to include an AT device if (1) >25% of participants reported the AT device as ‘I already have this’ or ‘I would use this, but I do not have it’ or (2) >25% of participants reported FDs that an AT device could compensate. The principal investigator also based her clinical expertise, which she had developed over 30 years as an occupational therapist specializing in working with older Latinos, to inform the selection of the final AT devices.

### 2.2. Phase II: Development of the Design Features and Prototype of the AT Guide Web App

After finalizing the content of the AT guide web app, a checklist was developed and used as a guide to design and evaluate the prototype. Professional application developers were employed to create the AT guide web app prototype using the Figma web design application.

#### 2.2.1. Measure

The researchers created the Accessibility Checklist for UX Designers tailored to the AT guide web app to assess design heuristics and ensure accessibility. Given the early stages of the user experience (UX) and user interface (UI) design of the web application, the research team decided to frame the application design around this checklist, which was developed using multiple sources. These sources included a customized adaptation of the Web Content Accessibility Guidelines (WCAG) [32], metrics based on checklists, guidelines, and other references from the Accessibility Guidelines Working Group [33,34], Princeton University [35], Google [36], and Team Treehouse [37], as well as senior-specific considerations such as cognitive decline and visual impairments, which are essential UX factors, as defined by Pokinko [38]. Additionally, established guidelines for designing accessible web apps for older adults were incorporated [39–43].

Ensuring that early-stage design factors align with senior accessibility needs lays a strong foundation for adherence to broader WCAG standards as development progresses. Our Accessibility Checklist for UX Designers organizes nineteen accessibility metrics into five key experience and design categories: text and wording (7 items), color and contrast (2 items), images and iconography (3 items), navigation (2 items), and design elements (5 items). Each item is assessed on the following scale: “Met,” “Not met,” or “Not applicable.” For the “Final Score”, each metric is assigned an equal value of 5.26%. This percentage was obtained by dividing 100% by 19 items, which gives approximately 5.26% per item (100% *÷* 19 *≈* 5.26%). This maintains an equal distribution of the total score, maximizing clarity and simplicity. Since the overall goal is to provide a clear assessment through the checklist, each item having an equal weight ensures that no single element disproportionately affects the overall accessibility score. The equal value of 5.26% assigned to each of the 19 accessibility metrics ensures a balanced assessment, reflecting the importance of all aspects of accessibility in alignment with WCAG standards.

#### 2.2.2. Procedures

The prototype design process followed a deliberate and research-driven approach conducted in two phases: (1) desk research and (2) design and development. Seven professional designers and application developers conducted the desk research phase. This phase was crucial in understanding the unique needs of this demographic and helped craft user personas that humanize the end user. The designers followed the guidelines of Redish and Chisnell [44] and came up with two personas that represented the application’s users. Designers encapsulated the personas in four attributes: age, ability, aptitude, and attitude. These attributes helped designers judge the need for support and training and the complexity of the features and functions that needed to be considered.

After the desk research phase, the designers took on the design and development phase, which started with low-fidelity sketching with pen and paper. Designing in this phase allowed quick and low-effort designing, allowing them to receive feedback from subject matter experts (the five occupational therapists researchers) and other designers in the team. The design process continued the feedback and iteration loop with mid- and high-fidelity designs, each being more detailed and closer to the end product.

As a final predevelopment process, designers used a visual design comparative exercise, assessing the UI and UX of four applications that have seniors as their target user (Lumosity: Brain Training, WebMD, Pillboxie, and Big Launcher). Taking into account the design heuristics, designers could visually inspect these applications and gather the best and worst practices that inspired the design of the AT guide web app. Afterward, we used the Accessibility Checklist for UX Designers to validate and confirm the prototype’s usability before proceeding to the development phase. In the last step of developing the AT guide web app, designers put together a working design prototype to be tested with participants’ users to ensure accessibility, design, and usability.

#### 2.2.3. Data Analysis

Descriptive statistics were used to analyze the frequency of the met, not met, and not applicable scales for the total score and each item of the Interface Accessibility Checklist. For the “Final Score”, each metric was valued at 5.26%. Designers set 75% as the acceptable final score for the accessibility audit. The audit score was only used to track adherence to the checklist and improve accessibility in the future by enhancing the final score.

### 2.3. Phase III: Usability Testing

In this phase, older Latinos who were digitally engaged, defined as participants who use a smartphone more than twice a week for purposes other than making phone calls, participated in one of the two focus groups conducted at the Medical Sciences Campus of the University of Puerto Rico. Older adults, classified as nondigitally engaged, were participants who used a smartphone two times a week or less. They participated in individual interviews at their homes. This phase aimed to investigate the design, utility, and usefulness of the MGAT prototype for older adults with physical FDs.

#### 2.3.1. Participants and Sampling

We used a nonprobability purposive sample to recruit participants for Phase III of this study. Four occupational therapy master’s degree students recruited the older adults using direct contact and snowball sampling for five weeks. The inclusion criteria were the following: (1) community-living Latino adults living in the community who are 65 years or older; (2) no requirement of supervision to perform their daily activities; (3) reported difficulties in one or more ADL or IADLs; and (4) have functional comprehension and verbal communication skills evidenced by a correct understanding of the purpose of the study and informed consent. Latinos younger than 65 years of age who received home healthcare services, were institutionalized, or were bedridden were excluded from this study. The sample population comprised seven older adults who participated in two focus groups (four in one group and three in the other) and seven older adults who participated in individual interviews with the student researchers. According to Nielsen [45], a small number of five participants is sufficient to identify usability problems.

The original research design included two focus groups, one comprised of digitally engaged participants and the other of nondigitally engaged participants. We aimed to have ten older adults in each focus group. However, we encountered recruitment challenges for the focus groups, including scheduling conflicts experienced by the digitally engaged participants and the need for transportation experienced by the nondigitally engaged participants. Alternative strategies to secure participation were to (1) schedule two focus groups for the digitally engaged participants that matched their availability and (2) add individual interviews for the digitally unengaged participants to ensure their participation.

#### 2.3.2. Measures

We developed the sociodemographic questionnaire used in the study to collect data on age, sex, education level, medical conditions, marital status, city of residence, number of people living with the participants, employment status, monthly income, sources of income, and healthcare plan. Additionally, our research team developed focus groups and individual interview guides to facilitate the discussion and collection of qualitative data from older adult participants to build the MGAT. Both guides used the same open-ended questions to obtain participants’ feedback on their experience using the application and their recommendations to improve its utility and usefulness for older adults. The questions included in both guides addressed the following seven facets of Karagianni’s Optimized Honeycomb Model [28]: (1) Findable—How easy was it to navigate or move within the MGAT?; (2) Accessible—Did you have any difficulties using the MGAT?; (3) Usable—How easy was it to use the MGAT? How confident did you feel using the application?; (4) Desirable—What did you like about the application? What did you not like about the application?; (5) Credible—Do you trust that the information provided was correct and accurate?; (6) Useful—How useful is the information in the application to facilitate your daily life activities?; (7) Valuable—What is this application’s value for people like you?

#### 2.3.3. Procedures

The sample of this study was divided into a digitally engaged group and a nondigitally engaged group. This allowed us to conduct two focus groups and seven individual interviews to gather information from two distinct groups based on their level of experience with mobile app technologies. The PI, who has expertise in qualitative research designs and data collection, developed a training manual, which was used to provide a comprehensive four-hour training session to the two occupational therapy master’s degree students who facilitated the focus groups. During the focus groups, the facilitators welcomed older adults. Then, they began the focus groups by introducing themselves, explaining the purpose of the meeting, and establishing ground rules for the discussion.

Subsequently, the facilitators provided scripted general information about AT and its benefits for the aging process and the self-management of FDs to improve participation in ADLs and IADLs. Subsequently, the AT guide web app was shown to participants by projection on a large screen TV monitor so that they could see its features adequately. This allowed the facilitators to provide a step-by-step explanation of accessing and navigating the application and exploring its content. The AT guide web app was then made available to participants on iPhone devices. The basic functionality of the iPhone was individually presented by four students with a master’s degree in occupational therapy so that the participants could use the tool freely and with maximum autonomy. Participants had time to learn and interact with the AT guide web app individually for an average of 40 min. The research team developed a task-based performance evaluation to assess the participant’s understanding of web application functions. This involved assigning specific tasks related to the navigation functionalities of the web app and observing how participants navigated and completed them. The instruction for this task was “Imagine that you have difficulty getting up from a chair and want to see what equipment could help you. Look for a piece of equipment you like and, as you look, tell me out loud what you are seeing and doing.” Subsequently, the data collectors observed the actions of the participants, the steps they took, and the level of ease or difficulty they experienced in completing the task. Errors were corrected until participants demonstrated the independent and correct use and navigation of the AT web app. The facilitators then asked their initial reactions and critical feedback guided by the focus group questions to obtain the user’s perspective on the web application.

During the individual interviews, the data collectors explained the purpose of the study. They showed the participants how to navigate the application using the same training and task-based performance evaluation procedures described for the focus groups. The participants could learn and interact with the AT guide web app individually for an average of 45 min. Afterward, the participants gave their perspectives on the use and characteristics of the application based on the interview-guiding questions.

#### 2.3.4. Data Analysis

Sociodemographic data were analyzed using descriptive statistics of central tendency: mean and standard deviations for the continuous variables and frequency and percentages for categorical variables. Microsoft Excel 2019 was used to perform the statistical analyses. Data from the focus groups and individual interviews were analyzed using a directed content analysis [46]. The responses were audio recorded, with the prior written consent of the participants, to increase the recollection of the data and were then transcribed verbatim by the researchers of this study after focus groups and individual interviews for subsequent analysis. Qualitative content analysis followed these steps: (1) develop the guiding framework based on the facets of the Honeycomb Model; (2) all data were read repeatedly and independently by research staff to achieve immersion and obtain a sense of and an understanding of the whole dataset; (3) all transcriptions were carefully reviewed, highlighting all text that identified meaning units according to the aim; (4) all highlighted text was condensed; (5) the condensed meaning units were deductively classified according to the predetermined facets of the Karagianni Optimized Honeycomb Model [28], wherever possible (text that could not be coded into one of these facets was coded with another label that captured the essence of the application problem or recommendation); (6) the condensed meaning units were coded; and (7) codes were inductively sorted to outline and label the subcategories within each facet. The Qualitative Data Analysis (QDA) Miner Lite (version 1.2) software was used as a data manager and organizer to support the coding process.

## 3. Results

### 3.1. Phase I

#### 3.1.1. AT Devices Included in the AT Guide Web App Based on Evidence of the Need for AT Devices

On a list of 50 AT devices in the Orellano-Colón et al. [30] study, the participants reported using 32 AT devices that compensated for physical function disabilities. The top three most frequently reported categories of AT devices that this sample used were medication (50%), personal hygiene (46%), and home safety (31%). This sample also reported 37 AT devices that compensated for physical function disabilities that they would use but did not have. The top three most frequently reported categories of AT devices they would use but did not have were (1) cooking (47%), (2) home tasks (37%), and (3) home safety (32%). Based on these results, 36 AT devices were included in the AT guide web app (Table 1). Five AT devices that did not reach the established frequency of >25% of the participants who reported the AT device as ‘I already have this’ or ‘I would use this but I do not have it’ were selected based on our clinical judgment. For example, the 3-in-1 commode, the wheelchair, and the scooter were included in the AT guide web app due to the critical role of these devices in increasing safety and occupational performance in functional mobility activities among older adults. On the contrary, 46.7% of the participants labelled the long-handle dustpan as ‘I already have this’. However, this device was not included in the AT guide web app because it is a commonly known cleaning device that does not meet the barrier of the lack of knowledge of AT devices found in our previous study [30].

#### 3.1.2. AT Devices Included in the AT Guide Web App Based on Physical Function Disabilities Reported Among Older Latinos

The results of Orellano-Colón et al. [31] revealed that the overall weighted prevalence of physical function disability using the T score among the study group was 58% (95% CI 36, 49%). The estimated prevalence of physical function disability was higher for instrumental activities for daily living (52%) compared to functional mobility (50%) and self-care (46%). Based on the results of the PROMIS Physical Function Short Form Individual Items (Table 2), the researchers identified 25 AT devices that could compensate for the physical function disabilities of this sample. Of these twenty-five AT devices, ten additional AT devices not identified in the Orellano-Colón et al. [31] study were selected to be included in the AT Guide web app. Clinical decisions were used to exclude the electric door opener due to its high cost.

#### 3.1.3. AT Devices Included in the Final Prototype AT Guide Web App

The research team chose 94 AT devices to include in the AT guide web app prototype. This amount of AT devices includes devices based on our previous studies, the variety of models available for some AT devices (such as different models of canes, elevated toilet seats, and bath seats), and additional types of AT devices that could compensate other physical FDs of older adults that were not evaluated by the PROMIS Physical Function Short Form, such as the variety of jar openers designed to address the difficulties older adults face in opening jars.

Table 3 reports the AT guide web app sections that address 14 modifiable barriers to the use of AT devices reported by Orellano-Colón et al. [16] and Orellano-Colón et al. [17]. For example, to address the barrier concerning the lack of functional needs, we included information on how the device could facilitate the performance of the activity of people with some level of physical difficulty in the benefits section of the AT device description. Additionally, the videos portray independent older adults using AT devices to convey that the AT device can be used by functionally abled older adults and not only older adults with significant limitations. To address the stigma barrier, the description section provides information on the availability of the AT device in different colors and designs to match each user’s self-image when available, fashionable, esthetic, and discrete options of AT devices that could conceal the design were selected in photos and videos. For example, the umbrella cane was included as an alternative to the regular cane, and the toilet base elevator was included as an alternative to the elevated toilet seat.

The barrier concerning the cost of the AT devices and services was addressed in several ways. First, the AT guide web app is free through its web address for information about AT devices and services. Second, low-cost AT devices were mainly included in the application. Third, devices classified as durable medical equipment had funding provision alternatives through healthcare medical plans.

**Figure 1.**
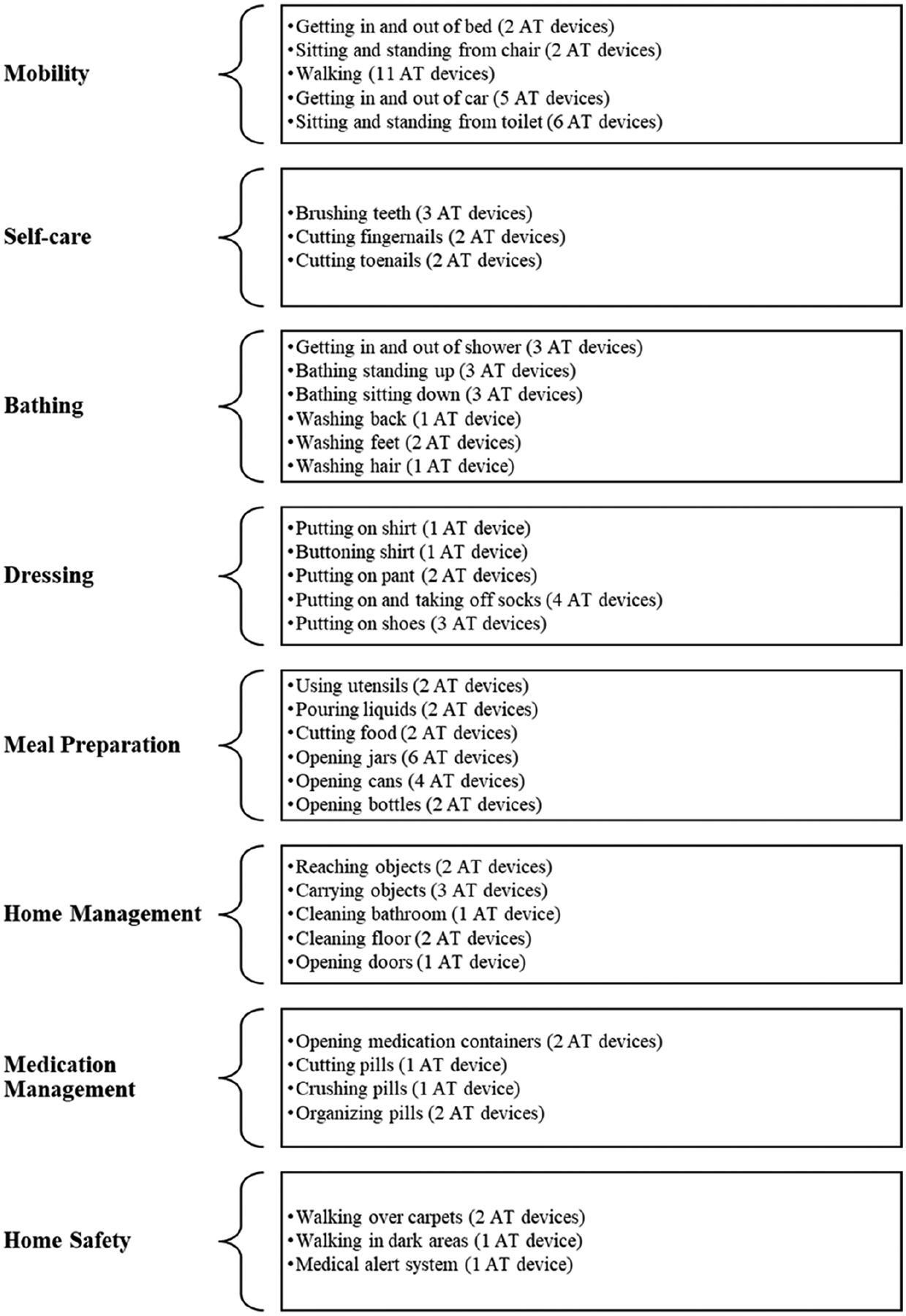
Content of the AT guide web app. The graphic lists the categories and subcategories of the daily activities included in the AT guide web app.

Finally, the first author drafted the web application instructions and detailed content and reviewed them with the research team over several sessions until consensus was achieved. Its content includes detailed information, photos, and videos of each AT device. The content of the AT guide web app is depicted in Figure 1. It consists of eight categories of daily activities: mobility, self-care, bathing, dressing, meal preparation, home management, medication management, and home safety. In addition, each category includes several tasks associated with each activity. For each task, the web app AT guide provides information on one or more AT devices that can facilitate user performance or safety during the task. The information provided for each AT device was developed in Spanish and included the name of the AT device in Spanish and English, a brief description of the AT device, the benefits (including the specifications of the AT device, proper device adjustment, and availability of a variety of colors and models when appropriate), considerations when acquiring and using the device to address user safety, AT providers, and the approximate cost range of the device. A disclaimer was prominently featured in each video at the beginning of the presentation. This disclaimer explicitly stated that the information provided was for educational purposes only and advised viewers to seek professional advice from an occupational or physical therapist for an assessment of their needs for the AT device.

### 3.2. Phase II

The AT guide web app prototype was created. The application was named the ‘Mi Guía de Asistencia Tecnológica” (MGAT; My Assistive Technology Guide) as the primary function of the application was to provide information about AT devices and resources to facilitate ADLs and IADLs of older adults with physical FDs. More information can be found at https://www.figma.com/proto/UcJ2xECanIxELPYr9pMxPq/RCM---Design-&-Prototype?page-id=593:2436&node-id=593-2753&viewport=−2020,1509,0.56&scaling=scale-down&starting-point-node-id=593:8348 (accessed on 21 October 2024). The MGAT is free and does not require a password or username. The web app format stored on a remote server and delivered over the Internet through a browser interface was selected instead of a native application due to its multiple benefits for older adults. First, web apps do not need to be installed, reducing the cognitive demands for their use. Second, web apps can be accessed through multiple browsers and platforms, such as desktop, laptop, or mobile, making them accessible to a broader population.

**Figure 2.**
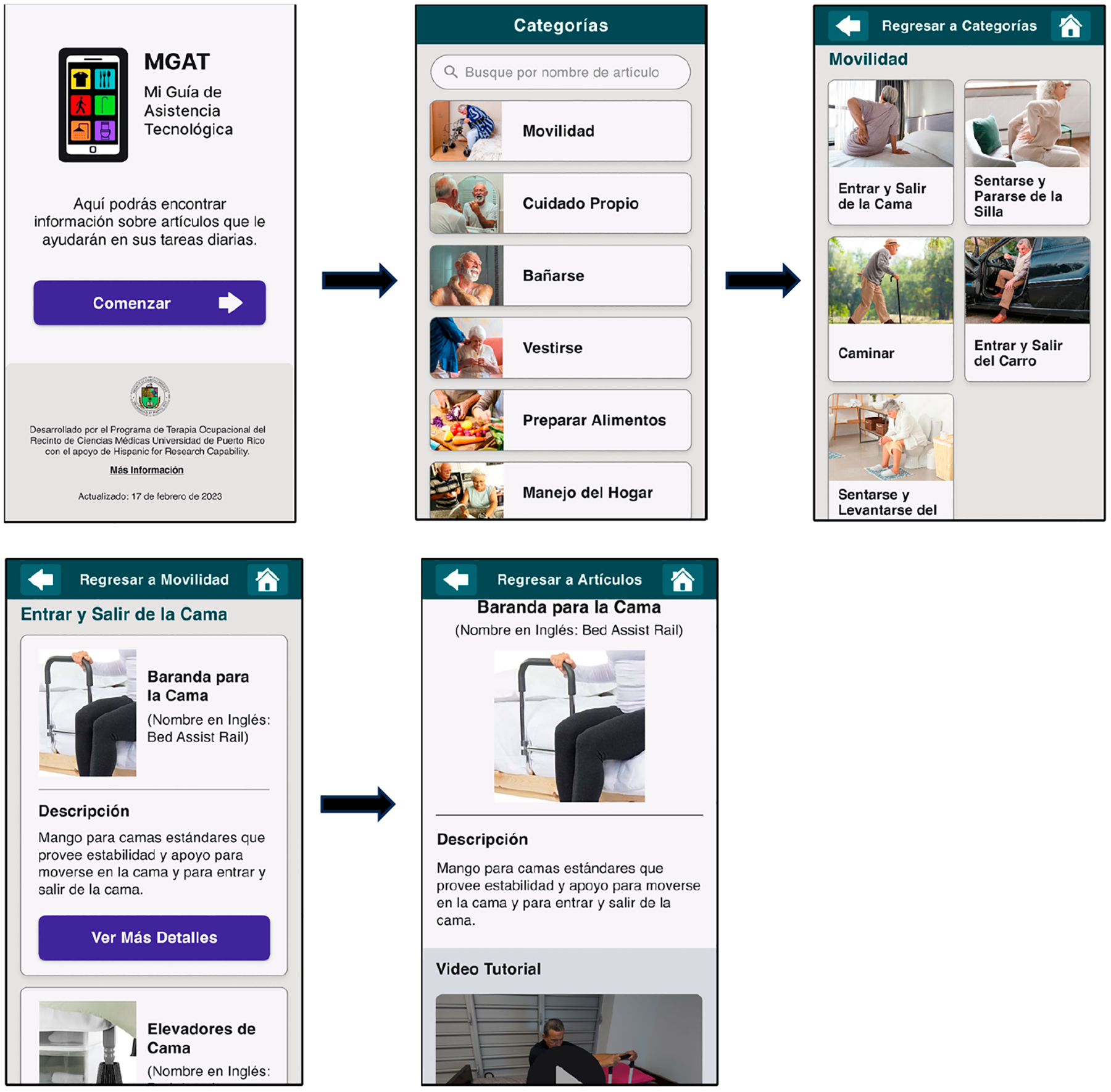
Screens of the web app. Screenshots depicting application navigation from the introductory screen to the home page, Categories of Activities Screen, Subcategories of Activities Screen, AT Device Options Screen, and the Final AT Device Screen.

A simple and engaging home page was created based on the Broderick et al. [47] framework for health literacy apps (Figure 2). The MGAT incorporates design options for older adults, such as user-friendly navigation features, touch and scroll-down options, back arrows and home icons, high-contrast colors, large buttons (52 pixels), and texts (16 pixels or more). It uses the Helvetica font, which is easy to read and contains simple language, a consistent use of buttons, limited options and information on each page, and visual graphics, including photos and videos of real older adults. Users can access relevant pictures, videos, and text information by touching or sliding the picture menus and printed information. The introductory page includes the MGAT logo, a brief description of the application’s objective, and a button to enter the home page. The home page consists of a simple graphic layout of AT photos and simple texts representing the AT categories of activities. On top of that, it includes a search feature to find AT devices by name. The user can easily access the home page using the home page icon on the top right of each screen and can go back to the previous page using the left arrow on the top left corner of the screen. An example of the organization for moving from the introductory page to the home page of daily activities, to the page for mobility-related activities, to the AT devices page, and the specific AT device page is shown in Figure 2. The final page on AT devices includes information related to the name, description, video, benefits, considerations, acquisition resources, and device cost.

Table 4 includes the results of the design audit based on the Accessibility Checklist for UX Designers adapted for the AT web application. Fourteen items from a total of nineteen met the accessibility criteria. Given an equal value of 5.26%, the frequency of met and not applicable metrics gave us a total acceptable final score of ~84.21%.

### 3.3. Phase III

On average, participants in the digitally engaged group were younger and had higher educational levels than those in the nondigitally engaged group. The nondigitally engaged group reported a higher prevalence of medical conditions than the digitally engaged group. Half of the participants were married. All nondigitally engaged participants lived with a significant other. In contrast, most of the digitally engaged participants lived alone. Only two of the participants were employed, while more than half were retired. The primary source of income for the total sample was Social Security. More than half of the participants were Medicare beneficiaries.

Of the twenty-nine invited individuals, fifteen did not agree to participate, eight accepted and confirmed their participation in the focus group, and seven accepted and confirmed their participation in the individual interview sessions. One of them did not attend the focus group. The fourteen participants who confirmed and were eligible were divided into digitally engaged (seven focus group participants) and nondigitally engaged (seven individual interview participants).

**Figure 3.**
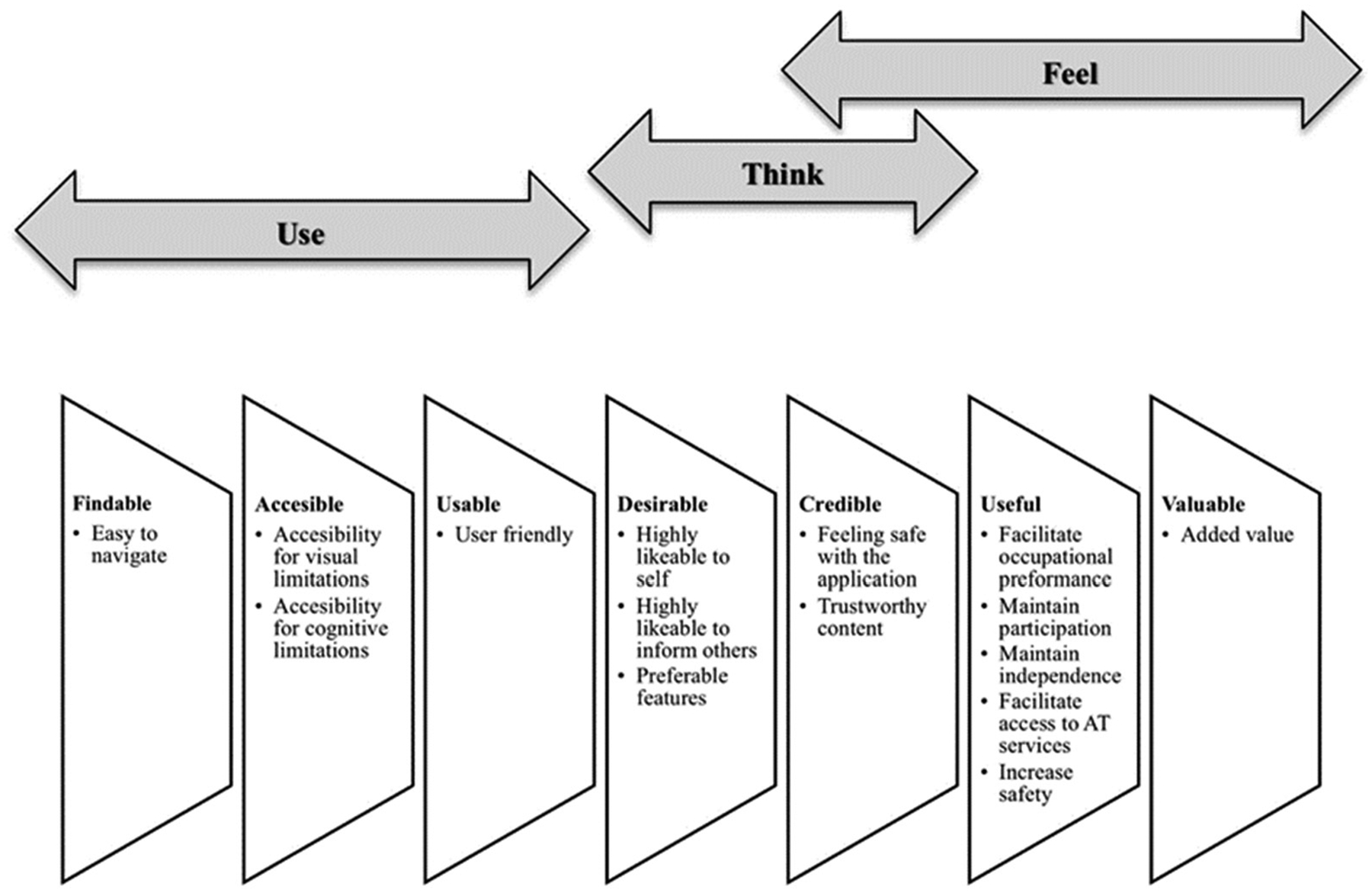
User experience themes and subthemes. The graphic depicts the user experience mapped onto the deductive themes and inductive subthemes based on the Optimized Honeycomb Model.

The results were categorized within Karagianni’s Optimized Honeycomb Model of Use, Think, and Feel dimensions (see Figure 3). We described each dimension’s corresponding Honeycomb Model facets and the subthemes that emerged from them. Within each facet, the subthemes were generated inductively. In total, 13 subthemes were derived, reflecting the opinions and suggestions of the participants. The main results found in this study indicate that the participants’ experience with how they think and feel about the MGAT was positive for older Latinos digitally engaged and nondigitally engaged. The participants also found the MGAT usable for them and others.

#### 3.3.1. Use

**Figure 4.**
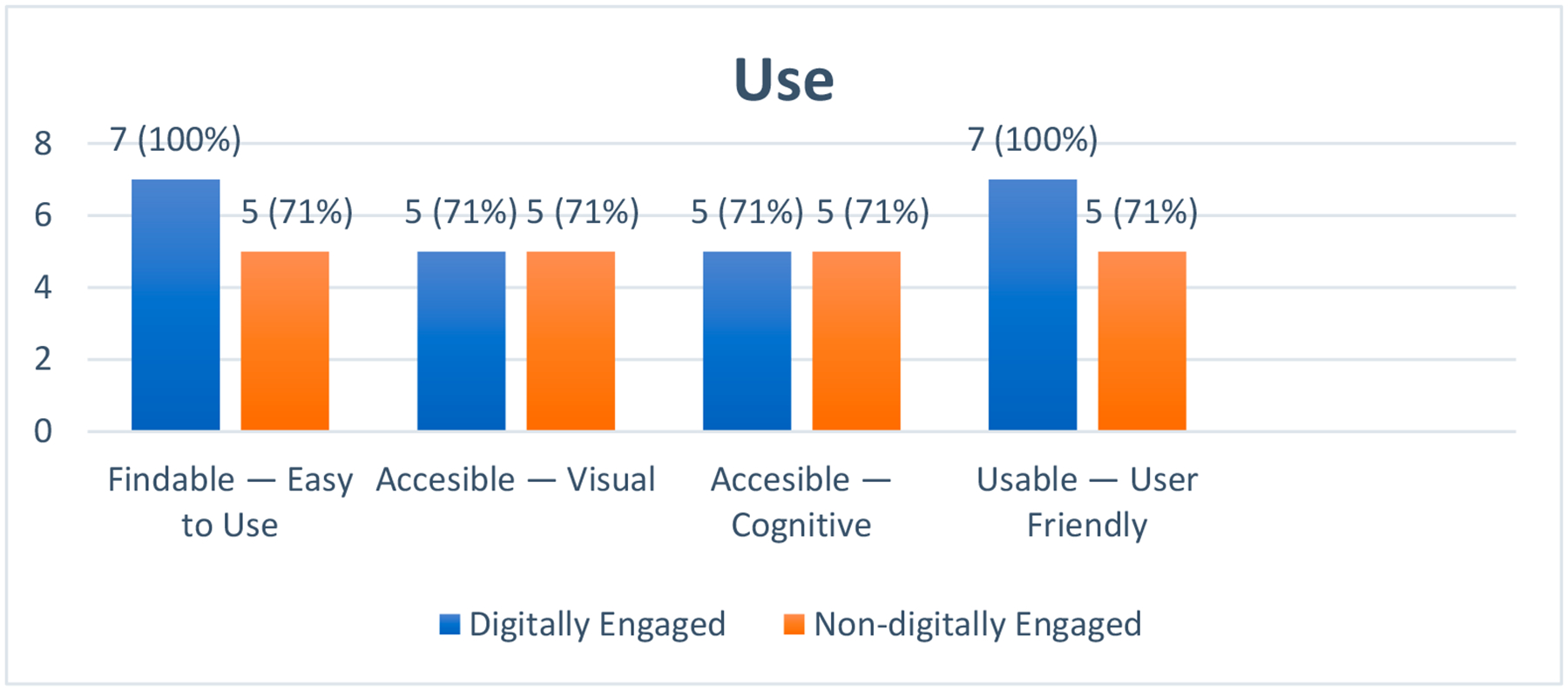
Participants experience within the ‘Use’ dimension of the Karagianni Optimized Honeycomb Model. The graphic depicts the user experience mapped onto the deductive ‘Use’ facets of ‘Findable’, ‘Accessible’, and ‘Usable’ and their corresponding inductive subthemes.

The theme of ‘Use’ refers to the practical aspects of how the MGAT is interacted with by users. It encompasses three critical subthemes: Findable, Visually accessible, Cognitively accessible, and Usable (see Figure 4).

The ‘Easy to Use’ subtheme emerged within the’ Findable’ dimension. Most of the participants indicated that the MGAT was simple and easy to navigate:

What I liked the most was that the categories that you chose are very clear and cover all areas of daily life for all of us, and they guide you; you do not have to be looking here, looking on the main page, like trying to guess; one can quickly identify the area where to find the information.

(Digitally engaged)

After individually exploring the MGAT, some participants experienced mild challenges, such as finding the button to access the last AT device details page. This challenge arose from the confusion that emerged from inconsistencies in the navigation gestures (sometimes tapping pictures and other times tapping text box). To solve this inconsistency, one of the participants recommended that “It would be beneficial that the information was accessed using the picture of the assistive device (instead of the text box)”.

Within the dimension ‘Accessible’, participants from both groups stated that the MGAT was ‘Visually Accessible’, as expressed by the following quote from a digitally engaged woman:

“(The MGAT) It has many visuals, is easy to see, and the letters are comfortable to read… In my case, I read with and without glasses, and I noticed that it is accessible to people with short vision and regular vision.”

Although the text was visually accessible, two participants recommended the following: “It should have an opportunity in which people can also amplify the text” (Digitally engaged). Recommendations were also made to enhance visual access to videos: “The recommendation is that you can see it completely (the video) because it is seen partially, there is always a missing part (when turning the mobile phone horizontally)” (Digitally engaged).

About the ‘Cognitively accessible’ subtheme, participants in both groups expressed that the language in the MGAT was simple and understandable. Most of the participants also found the videos accessible. However, to enhance the cognitive access of the MGAT, various participants in both groups agreed that videos should include audio descriptions of instructions for using AT devices.

Finally, within the facet of ‘Usable’, the ‘User-friendly’ subtheme emerged, as the MGAT was easy to operate, as stated by the following participant:

It is so easy (to use). It cannot be easier… Many times, they ask for many questions and passwords and things, and this does not have any of them. You go direct, and everything is clear, everything is good … They do not ask me anything complicated.

(Digitally engaged).

#### 3.3.2. Think

**Figure 5.**
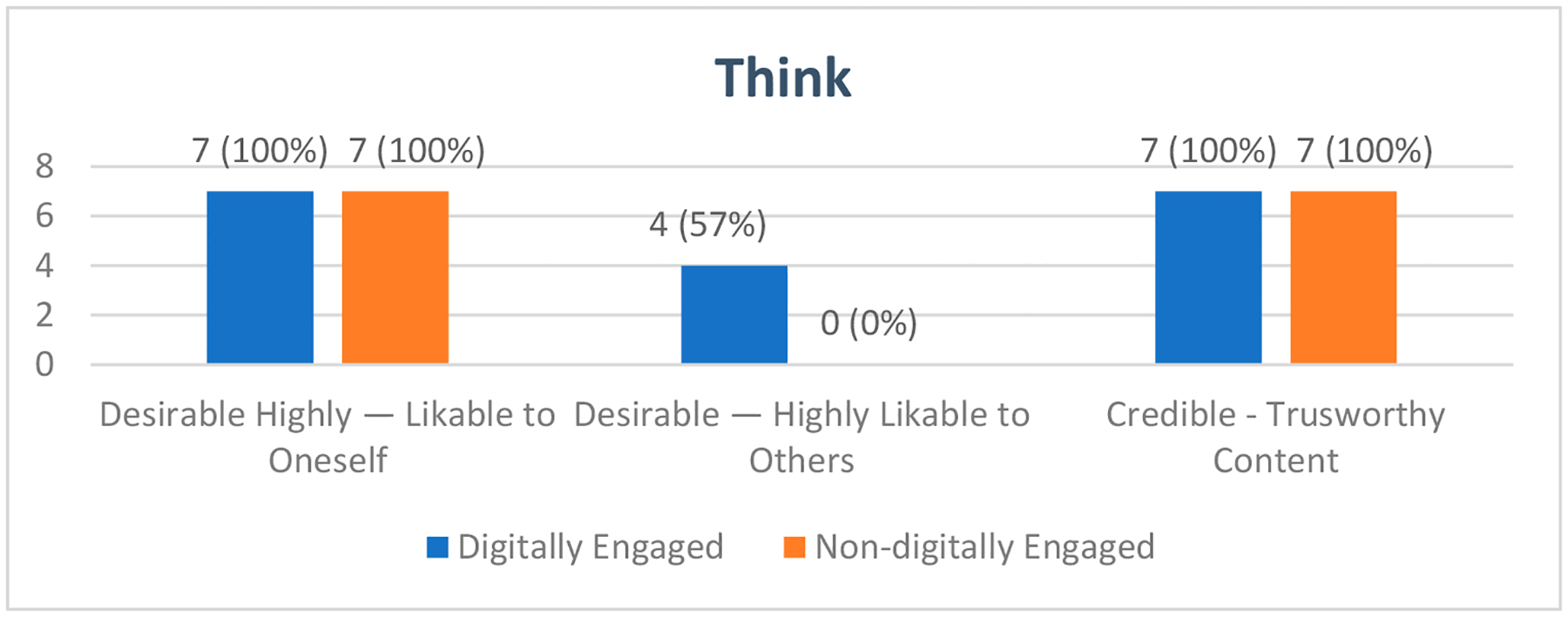
Participants experience within the ‘Think’ dimension of Karagianni’s Optimized Honeycomb Model. The graphic depicts the user experience mapped onto the deductive ‘Think’ facets of ‘Desirable’ and ‘Credible’ and their corresponding inductive subthemes.

The theme of ‘Think’ revolves around users’ perceptions and attitudes toward the MGAT. It addresses three key subthemes: Desirable—Highly likable to oneself; Desirable Highly likable to inform others; and Credible—Trustworthy content (see Figure 5).

The ‘Desirable’ facet subtheme of ‘Highly likable to oneself’ was evidenced by the interest of the participants in exploring all the AT devices in the MGAT and expressions such as “it surprised me”, “I loved it”, “I liked it all”, and “it met my expectations”.

The MGAT was also attractive because it allowed participants to advise others about AT devices, as represented in the subtheme “Highly likable to inform others”.

The (MGAT) is not only a resource for us in the third or fourth age. It is an aspect that must be given to children, adolescents, and older adults. Because I have this here at my house (grab bars), it is not that I am old, because when you guys also enter the bathtub, especially children, if there is a tube, it will give you security… So, it is not just a resource for the third or fourth age. But it is an educational resource for all ages.

(Digitally engaged)

Within the ‘Credible’ facet, all participants agreed that the MGAT provided ‘Trustworthy content’ and accurate information because a trustworthy institution developed it. Two digitally engaged participants talked about the trustworthiness of the MGAT based on its professional design characteristics.

Its colors convey credibility. They are green colors used by nurses; black is standard, and white is the background. In other words, it’s serious; it’s not that they tried to make it look nice. This is professional; these are the colors of medical science. And this gives one a sense of tranquility—that I am seeing something fine. This is not nice; this is serious.

(Digitally engaged)

Nevertheless, various participants from the digitally engaged group gave valuable recommendations on improving the MGAT’s credibility.

I imagine that at some point, those who made the application will be on the home page because that gives confidence to the person that it is not a commercial thing, that they are not trying to, you know, sell you something. Let it be seen that there is an investigation there, that there are professional people who work on it, and that it is updated. Putting dates, such as the updated date of the apps, is very important. When was the last? Because things change every day, even teams. That is updated on 3 December 2022, prepared by Occupational Therapy Master’s Degree students, something like that, because that gives confidence when using the application.

#### 3.3.3. Feel

**Figure 6.**
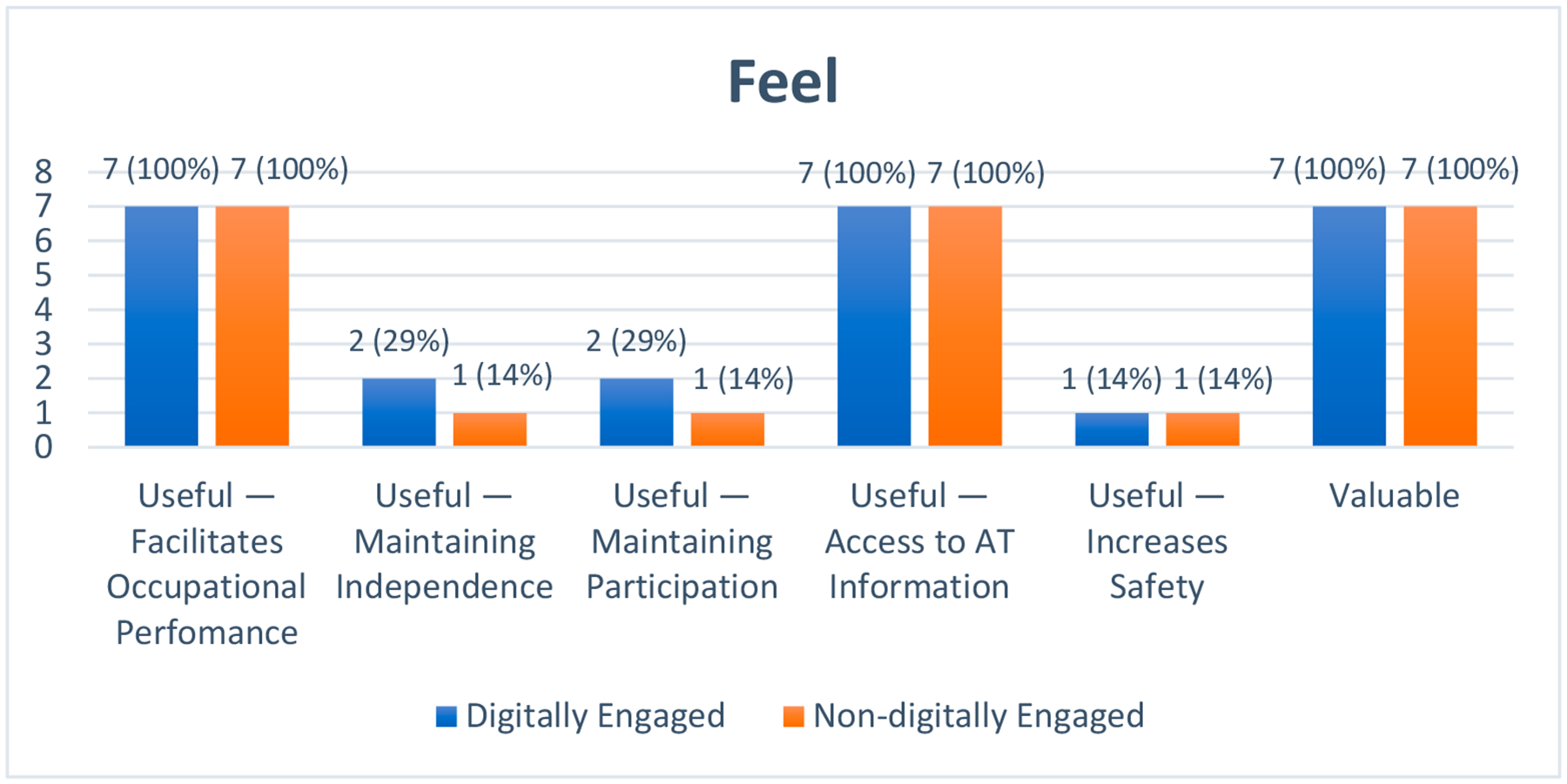
Participants’ experience within the ‘Feel’ dimension of Karagianni’s Optimized Honeycomb Model. The graphic depicts the users’ experience mapped onto the deductive ‘Feel’ facets of ‘Useful’ and ‘Valuable’ and their corresponding inductive subthemes.

The theme of ‘Feel’ focuses on users’ emotional responses and subjective experiences when interacting with the MGAT. It encompasses six primary subthemes: Useful—Facilitates occupational performance; Useful—Maintains independence; Useful—Maintains participation; Useful—Access to AT information; Useful—Increases safety; and Valuable (see Figure 6).

‘Useful’ was the facet with the most references cited by the participants. They agreed that the MGAT was beneficial for different purposes. Specifically, under the ‘Facilitate occupational performance’ subtheme, they highlighted how the MGAT helped to provide information that enhanced their understanding of different AT devices available to support activities of daily living ADLs and IADLs. Additionally, while more digitally engaged participants expressed that they currently did not have a functional need for AT devices, they perceived the MGAT as valuable in offering insights into a wide range of AT devices that could potentially assist them in overcoming future occupational limitations later in life.

One is already reaching an age where you will need something in the future. Notice that everything that is there was unknown to us, that we did not know existed. Now we know that it exists… I already know that when I need something, I can go there and buy it, and it will help me.

(Digitally engaged)

Digitally engaged participants also discussed the benefit of knowing about AT devices that could inform others or provide care in their daily activities.

I learned a lot because there were instruments and things that we could have used with my mom when she was already sick enough that we didn’t know about. I told Adriana: “Wow, imagine if we had known about this (transfer tub bench)”. You know, it guides you well on things you can use to help not only you but also the people you care for.

(Digitally engaged)

On the contrary, more participants in the nondigitally engaged group talked about how the new knowledge gained from the MGAT could be presently helpful for themselves: “It is useful because you do things correctly and it can help with your difficulties… we know what things need to be changed or what one can buy to be well. Therefore, everything becomes easier”. (Nondigitally engaged)

The usefulness of the MGAT in maintaining older adults’ performance in daily activities formed the subtheme ‘Maintains independence’.

The application allows you to have more independence. For example, if I have a seat in the bathroom and have difficulty using the bathroom to bathe… I have some attachments; it will be easier, and I won’t depend on someone else to help me. That is very useful because it gives you independence. That is self-help.

(Digitally engaged)

Similarly, the ‘Maintains participation’ subtheme expresses the participant’s reflections about the potential of the MGAT to inform about AT devices that can maintain their participation in meaningful occupations, thus delaying the need for institutionalization.

(The MGAT) includes many things that we do not imagine exist, and it is very good that this is disseminated because many people think that we have to go to home care and, no, not yet. There are many things that you can use and still ride on the street. We always have the option of being able to extend the possibility of remaining independent.

(Digitally engaged, man)

The ‘Access to AT information’ subtheme describes the usefulness of the MGAT in providing access to information on any service that directly assists older adults in selecting, acquiring, or using an AT device. The most frequently mentioned benefit was the potential of the MGAT to increase user access to information about the variety of AT devices for ADLs and IADLs. The participants also appreciated the details about acquiring AT devices and the associated costs, enabling them to make informed decisions.

Information is very important because it lets us see where we will get what we need for our problems. Because they (older adults with FDs) often don’t want to accept it. Why is this happening to me? But there comes a time when we need to realize it. I must put the grab bars in the bathroom; where will I get them? What equipment am I going to put in the toilet? Where can I get it? The price, because when seeing such a device at such a cost, well, you know, we tend to look for the cheapest for our income. And that gives us more self-help and security.

(Digitally engaged)

One participant in each group expresses the ‘Increase safety’ subtheme. It refers to the views of participants on how using AT devices can potentially increase safety in the performance of daily activities: “It is useful because it helps avoid accidents” (Nondigitally engaged).

The final ‘Value’ facet comprises the six facets surrounding the MGAT and illustrates the added value delivered by the MGAT to the user and to advance the mission. All participants considered the MGAT valuable to them or others. They expressed their experiences with having FDs to carry out their daily activities. They identified the value of learning about AT devices to improve their performance and maintain independence, particularly for older adults living alone.

Well, it is very valuable (the MGAT), and it is very useful because, for a little while, it has made my life easier, and I don’t have to depend on anyone. I live alone and can manage things at home and outside the house… Without anyone’s help.

(Digitally engaged)

In addition, the general value of the MGAT was discussed in terms of its scope to promote and improve the quality of life of people currently faced with physical FDs: “It has immense value… Because if that thing on the bed (bed handle) helps me get up, wonderful” (Nondigitally engaged). Furthermore, those participants in the digitally engaged group who perceived a lack of functional need to use AT devices could see the benefit of the MGAT in improving the quality of life of their significant others experiencing FDs.

Well, that is not the case for me right now (MGAT has no value) … but in my mother’s case, yes, it is a lot. She can significantly improve her quality of life. What happens to her is that it hurts (her back). For example, this thing that rotates in the car (car pivot seat) does not take away the pain, but it hurts less time, or it hurts less because you don’t have to make as much effort and it consumes less energy, which gives you more strength to get up.

(Digitally engaged).

Additionally, two participants in the digitally engaged group expressed the potential of MGAT videos to help older adults psychologically adjust to their physical FDs: “Even when the person is in an attitude of denial (of having a physical FD), by being so graphic (MGAT videos), the moment comes when the person says, this is going to facilitate my process”.

## 4. Discussion

The purpose of this study was to develop an AT guide web app (MGAT) for older Latinos living in the community with physical FDs in ADLs and IADLs. The development and usability testing of the MGAT resulted in a findable, usable, accessible, credible, desirable, useful, and valuable web app for older adults to access information about AT devices and services in Puerto Rico.

The main results of the usability phase showed that the perceived usefulness of the MGAT was different for the digitally engaged and nondigitally engaged groups. While more participants in the nondigitally engaged group perceived the benefit of the MGAT in knowing about AT devices that could enhance their current occupational performance, more participants in the digitally engaged group saw the benefits of the MGAT in learning about AT devices that they could use in the future as they age or about devices that could facilitate caregiving for their significant others. Differences in the characteristics of both groups could explain this result. Since the nondigitally engaged group was older and reported more medical conditions, this group probably experienced higher levels of physical FD [1].

To our knowledge, the UX Honeycomb Model (original and optimized versions) has been used in two other qualitative studies in health-related research to conduct development and usability testing with older adults. These studies targeted different areas, such as patients with glaucoma using a prototype to learn about their conditions [48] and the development of a smartphone self-test application for balance and leg strength in collaboration between older adults and the research team [49]. The researchers’ analysis in the current study, using Karagianni’s Optimized Honeycomb Model, yielded findings similar to those of the previous qualitative studies. In the ‘Use’ category, most participants found that the MGAT was easy to navigate, was simple to use, included clearly categorized information, and was visually and cognitively accessible. The other two studies that used the UX Honeycomb or Optimized Honeycomb Model reported similar results with ‘Use’. In the application of the balance self-test, the need for clear instructions, adaptations for vision, hearing, and cognitive impairments, and straightforward and logical organization of information was expressed [49]. Patients with glaucoma expressed the need to limit content [48]. However, two nondigitally engaged participants required assistance scrolling through the MGAT content pages after receiving training, which was consistent with the participant’s lack of smartphone skills. Moreover, digitally and nondigitally engaged groups tapped the wrong button to access the AT device details page using a text button instead of a picture button, which was consistently used for navigation through the previous MGAT pages. These results highlight the importance of maintaining consistency in the navigation system used for older adults, as recommended in previous guidelines [50,51].

Within the ‘Feel’ category, some noticeable similarities were that older adults were interested in learning new things. Those participants in this study, independent of being digitally or nondigitally engaged, wanted to be MGAT end users and were interested in learning about available AT devices and services to overcome physical FDs. Other studies reported that older adults were interested in learning a new balance self-test [49] and that knowledge was empowering in patients with glaucoma [48]. Furthermore, in ‘Feel’, the participants’ confidence in the credibility of the information provided by the MGAT was supported by the development of the web app by a higher educational institution in Puerto Rico. Similarly, Fearns et al. [48] found that the involvement of appropriately qualified professionals in developing an application was critical to the credibility of guidelines for glaucoma patients.

In the category ‘Think’, regardless of whether they are digitally or nondigitally engaged, the participants expressed the potential of the MGAT to facilitate their occupational performance, independence, and participation in daily activities today or in the future, increase their access to AT services, and ensure safety in the performance of daily living activities. Other studies described usefulness as the need to apply balance self-tests [49] and the dissemination of guidelines for users in the glaucoma study [43]. An added value of the MGAT was its potential to help older adults adjust to their physical FDs by observing videos of older adults using mainstream technologies. However, none of the other studies used the UX Honeycomb Model as a framework related to disability adjustment.

The participants’ recommendations for improving the MGAT were mainly associated with the ‘Use’ category. Although the MGAT text was designed following current heuristic design guidelines for older adults and was considered easy to see by both the digitally and nondigitally engaged groups, participants recommended including a more extensive text feature to accommodate future vision loss. This finding shows the importance of including customizable features to accommodate older adult’s current and future visual needs. Both groups also recommended the inclusion of audio descriptions of the videos. Given the positive effect of providing video instructions in the self-test application for the balance study [49], future MGAT redesign activities must address adding audio instructions to the videos.

To the authors’ knowledge, this is the first study to develop and test the usability of a web app that provides information about ADD for daily living activities to overcome limitations of the physical function of older adults. Although the app itself is the primary contribution, the originality of the MGAT prototype lies in its specific focus on providing detailed AT information tailored to older adults with physical FDs, which is a unique target population compared to existing AT information resources, such as the Atvisor.ai [26]. Atvisor.ai is a general artificial intelligence platform that supports AT assessments and decision-making processes without a specific focus on older adults with physical FDs. Additionally, the MGAT prototype incorporates novel AT information, such as the particular benefits of each AT device, essential considerations that must be taken before acquiring each device, and videos of older adults who demonstrate the use of each AT device, which are unique features not found in the Atvisor.ai platform. The visual approach of videos helps users to better understand how AT devices can be integrated into their daily lives, a key consideration for the MGAT’s target audience of older adults with physical FDs.

There is a gap in rehabilitation services for older Latinos living in the community with FDs. New methods for managing FDs are needed, as occupational and physical therapy services are limited. AT devices could be a step forward in helping older adults improve their function in activities and occupations of everyday life. A newly and empirically developed web app for AT, developed by occupational therapists, could allow healthcare professionals, older adults, and their significant others to access credible and practical information about AT devices to overcome the limitations of older adults with physical FDs in ADLs and IADLs. A free AT web app might also address the predominant barrier to using AT devices among older Latinos living in Puerto Rico with physical FDs: the lack of access to information about AT devices. Since knowledge of AT is an essential first step in acquiring and using AT devices, the MGAT holds promise in addressing disparities in independent living disabilities among older Latinos.

### Limitations

The limitations of the MGAT prototype at this stage reside in its inability to support key accessibility features of any access platform, such as computers, tablets, or smartphones. Furthermore, the MGAT does not provide recommendations for assistive technology decision-making. It is limited to providing information about AT for older adults with FDs in activities of daily living and instrumental activities of daily living. Finally, the AT devices included in the MGAT were selected from samples of independent-living older Latinos living in Puerto Rico with mild to moderate physical FDs in daily living and instrumental activities of daily living. Therefore, the usefulness of the MGAT cannot be generalized to older adults with more severe physical function disabilities or with sensory or cognitive disabilities. These subgroups of older adults have different AT device needs not addressed by the MGAT prototype.

## 5. Conclusions

This study found that the evidence-based background for developing the MGAT content, the checklist used for this study, and the Optimized Honeycomb Model for user experience were practical for designing and testing the usability of the MGAT for older adults. Building the content from evidence-based barriers to using AT devices and physical function disabilities reported among older Latinos increases the potential of the MGAT to be acceptable, usable, and valuable for older Latinos. Moreover, AT web app design for digitally as well as nondigitally engaged older adults should consider the following features: free of cost, does not need a password, user-friendly navigation features (i.e., touch and scroll down options, back arrows, home icons, high contrast colors, large buttons, easy to read larger fonts, simple language, consistent use of buttons, a limited number of options and information on each page), and visual graphics including photos and videos of real older adults. The results from the analysis indicate that participants had positive experiences with the MGAT. Additionally, several recommendations were provided to improve the use, accessibility, and credibility of the MGAT. These findings serve as an essential foundation for developing appropriate and efficient AT information strategies, using this technology as a first step to improve AT adoption and use among older adults. Although the MGAT prototype was developed based on the needs of older adults in Puerto Rico, the evidence-based approach and user-centered design techniques used in its development could be applied to create similar AT information resources tailored to older adults of other nationalities and cultural backgrounds. This suggests the potential for the MGAT’s results to be generalized to a broader population of older adults with similar functional needs, pending further research and validation. Future research must test the reliability and validity of the adapted Accessibility Checklist for UX Designers with application experts and end users. Furthermore, future work must assess the feasibility of using the MGAT to improve older adults’ knowledge, attitudes, and behaviors toward using AT.

## Figures and Tables

**Table 1.** Assistive technology (AT) devices included in the AT guide web app based on older Latino’s AT needs [29].

AT Devices	I Already Have This	I Would Use This, but Do Not Have It	Inclusion in the AT Guide Web App (Yes/No)
Home tasks			
Jar openers	15 (25%)	35 (58%)	Yes
Electric can opener	24 (40%)	21 (35%)	Yes
Reacher	23 (38.3%)	28 (46%)	Yes
Long-handle duster	10 (16.7%)	10 (16.7%)	No
Laundry basket with wheels	10 (16.7%)	30 (50%)	Yes
Long-handle dustpan	28.0 (46.7%)	17 (28%)	No
High stool with long handle	15 (25%)	11 (18.3%)	Yes
Long-handle cleaning brush	7 (11.7%)	26 (43.3%)	Yes
Handle for carrier bags	1 (1.7%)	15 (25%)	No
Cooking			
Built-up handles for utensils	12 (20%)	27 (45.0%)	Yes
Home safety/security			
Adhesive tape to stabilize rugs	5 (8.3%)	27 (45.0%)	Yes
Grab bars	40 (66.7%)	7 (11.7%)	Yes
Night light	37 (61.7%)	5 (8.3%)	Yes
Non-slip mat	27 (45%)	17 (28.3%)	Yes
Non-slip rubber	1 (1.7%)	29 (48.3%)	Yes
Home accessibility			
Rail for bed	18 (30%)	11 (18.3%)	Yes
Bed or chair lifts	1 (1.7%)	22 (36.7%)	Yes
Lever knobs	18 (30%)	9 (15%)	Yes
Emergency alert system	13 (21.7%)	26 (43.3%)	Yes
Seat lift	0 (0.0%)	34 (56.7%)	Yes
Personal hygiene			
Nail clipper with enlarged handle and magnifier	4 (6.7%)	17 (28.3%)	Yes
Tub bench	28 (46.7%)	15 (25.0%)	Yes
Long-handle sponge	40 (66.7%)	21 (35.0%)	Yes
Hand shower	40 (66.7%)	13 (21.7%)	Yes
Commode 3-in-1	6 (10%)	9 (15%)	Yes
Medication			
Pill organizers	52 (86.7%)	1 (1.7%)	Yes
Medication reminders	8 (13.3%)	25 (41.7%)	Yes
Dressing			
Dressing stick	3 (5%)	17 (28.3%)	Yes
Long-handle shoehorn	13 (21.7%)	22 (36.7%)	Yes
Sock aid	1 (1.7%)	22 (36.7%)	Yes
Button hook	0 (0.0%)	15 (25%)	Yes
Mobility			
Cane	43 (71.1%)	1 (1.7%)	Yes
Walker	19 (31.7%)	8 (13.3%)	Yes
Wheelchair	7 (11.7%)	3 (5.5%)	Yes
Shopping cart on wheels	9 (15%)	29 (48.3%)	Yes
Scooter	0 (0.0%)	9 (15.0%)	Yes
Toilet use			
Raised toilet seat	2 (3.3%)	6 (10.0%)	Yes
Raised toilet base	3 (5%)	13 (21.7%)	Yes
Rails around toilet	4 (6.7%)	19 (31.7%)	Yes
Others			
Remote controls for electrical equipment	0 (0.0%)	16 (26.7%)	No

Note. AT = assistive technology.

**Table 2.** Assistive technology devices included in the assistive technology guide web app based on physical function disabilities reported among older Latinos [30].

Daily Activities	Web App	AT Devices That Could Compensate for the Disability	Inclusion in the AT Guide Web App (Yes/No)
Instrumental Activities of Daily Living			
Household chores (i.e., vacuuming or yard work)	94 (45%)	Lightweight vacuum	Yes
		Garden seat	Yes
Pushing open a heavy door	98 (46%)	Automatic door opener	No
Lifting and carrying grocery bags	113 (54%)	Grocery cart with wheels	Yes
Holding a plate full of food	45 (21%)	Kitchen cart with wheels	Yes
Participating in vigorous activities (running, lifting heavy objects, or participating in strenuous sports)	174 (82%)	Bottle pouring equipment	Yes
		Laundry basket with wheels	Yes
Bending, kneeling, or stooping	137 (65%)	Reacher	Yes
Self-care			
Washing back	81 (38%)	Long-handle shower sponge	Yes
Drying back with towel	53 (25%)	Bathrobe	No
Washing and drying body	42 (20%)	Long handle brush for feet	Yes
		Sponge for toes	Yes
Dressing, including tying shoelaces and wearing buttons	62 (29%)	Dressing stick	Yes
		Sock aid	Yes
		Elastic shoelaces	Yes
		Long-handle shoehorn	Yes
		Button hook	Yes
Squeezing a tube of toothpaste	37 (18%)	Toothpaste dispenser	No
Shampooing hair	34 (16%)	Hair washer	Yes
Functional mobility			
Sitting on the edge of a bed	57 (27%)	Bed rail	Yes
Getting in and out of a car	87 (41%)	Handle for car	Yes
Running a short distance	160 (76%)	Scooter	Yes
Getting to and from the toilet	52 (25%)	Toilet seat elevator	Yes
Transferring from a bed to a chair and back	63 (30%)	Bed rail	Yes
		Seat elevator	Yes
Walking more than a mile	127 (60%)	Cane	Yes
		Rolling walker	Yes
Climbing one flight of stairs	115 (54%)	Cane for climbing stairs	Yes

Note. AT = assistive technology.

**Table 3.** Assistive technology (AT) guide web app sections that address the barriers to using AT devices.

Barriers to the Use of AT Devices	Content (Description, Cost, Acquisition Resources, Benefits, and Considerations)	Picture	Video
Person-related			
Perceived lack of functional need	✓ (Benefits)		✓
Stigma	✓ (Description)	✓	✓
Fear			✓
Safety concerns	✓ (Description and considerations)		✓
Perceived complexity			✓
Technology-related			
Lack of availability	✓ (Acquisition resources)		
Cost	✓ (Cost)		✓
Poor performance	✓ (Considerations)		
Discomfort	✓ (Considerations)		
Unattractive appearance	✓ (Description)	✓	✓
Environment-related			
Limited access to information about AT devices, skills, and resources	✓ (All sections)	✓	✓
Limited access to AT services and funded provision	✓ (Acquisition resources)		
Lack of access to the physical environment	✓ (Description and Considerations)	✓	✓
Gender-related			
Gender differences	✓ (Benefits)	✓	

Note. AT = assistive technology. The ✓ symbol indicates that the corresponding barrier to using AT devices has been addressed by the web app content, picture, or video.

**Table 4.** Results of the design audit based on the 19 items of the adapted Accessibility Checklist for UX Designers.

Accessibility Categories	Met	Not Met
Text and Wording	6 out of 7	0 out of 7
Color and Contrast	2 out of 2	0 out of 2
Images and Iconography	0 out of 3	2 out of 3
Navigation	2 out of 2	0 out of 2
Design and Elements	4 out of 5	1 out of 5

## Data Availability

The data presented in this study are available upon request from the corresponding author due to restrictions. The data are not publicly available.
